# Hepatic Peliosis due to Azathioprine in a Pediatric Kidney Transplant Recipient

**DOI:** 10.1155/crhe/8752052

**Published:** 2025-11-14

**Authors:** Antoine Mouche, Cyrielle Parmentier, Marion Almes, Charlotte Mussini, Tim Ulinski, Jean-Daniel Delbet

**Affiliations:** ^1^Pediatric Nephrology Unit, Armand Trousseau Hospital, DMU Origyne, APHP, Paris, France; ^2^Sorbonne University, Paris, France; ^3^Pediatric Hepatology Unit, Kremlin Bicetre Hospital, APHP, Kremlin Bicetre, France; ^4^Anatomopathology Unit, Kremlin Bicetre Hospital, APHP, Kremlin Bicetre, France

## Abstract

Azathioprine, an antimetabolite drug interfering with purines synthesis, is one of the immunosuppressive maintenance therapy used in solid organ transplant or dysimmune disease. We report the case of a 5-year-old-girl with Stage 5 chronic kidney disease, who developed hepatic peliosis due to azathioprine used 9 months after kidney transplant.

## 1. Case Study

A girl with Stage 5 chronic kidney disease due to Denys–Drash syndrome (WT1 mutation) received a kidney transplant from a deceased donor at the age of 5 years. She was treated on hemodialysis since the age of 18 months. Preventive bilateral nephrectomy was performed at 2 years.

Cross match (performed by LCT technique) was positive on IgM with a previous serum. Therefore, immunosuppressive induction therapy consisted of the association of methylprednisolone, anti-human thymocyte immunoglobulin preparation, mycophenolate mofetil (MMF), and tacrolimus.

Immunosuppression maintenance therapy was an association of tacrolimus and MMF. Seven months posttransplant, MMF was switched to azathioprine because of persistent diarrhea attributed to this treatment.

Two months after (9 months posttransplant), the patient presented to our emergency department with fever, vomiting, and diarrhea. On physical examination, the patient had a temperature of 38.1°C, no hemodynamic disorders (pulse rate 131/min, blood pressure of 109/48 mmHg). Mild abdominal distension was observed with tenderness but no guarding. A distended bladder was clinically palpable. Bowel sounds were present.

Laboratory tests revealed a bicytopenia (hemoglobin 8.6 g/dL and leukocytopenia 1.75 G/L with neutropenia of 0.53 G/L). Platelets were normal at 304 G/L. There was an inflammatory syndrome (CRP 165 mg/L), acute renal failure (urea 20 mmol/L, serum creatinine 387 μmol/L for a basal value of 50 μmol/L), hyponatremia of 120 mmol/L, and hypokalemia of 3.1 mmol/L. Bicarbonates were at 18 mmol/L. Liver function was normal, aspartate transaminase (AST) and gamma-glutamyltransferase (GGT) were slightly increased at, respectively, 36 and 47 UI/L, and direct bilirubin was 9 μmol/L (normal < 6 μmol/L). Alkaline phosphatase was low at 89 μmol/L. Residual tacrolimus level was 10.2 ng/mL (daily dose 0.6 mg/12 h, i.e., 0.05 mg/kg pe day). 6-Thioguanine nucleotide (6-TGN) value was 2855 pmol/8 × 10∗8 RBC (N 200–600) and 6-Mercaptopurine nucleotide (6-MMP) was 889 pmol/8 × ∗8 RBC (*N* < 5800) (daily dose 50 mg/d i.e. 2.4 mg/kg per day). Donor specific antigen DR 52 was positive at a stable low MFI rate of 682.

**Figure 1:**
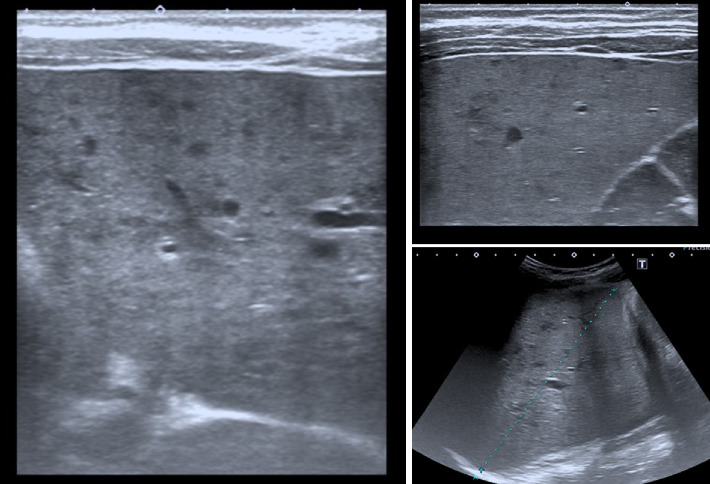
Abdominal ultrasound. This ultrasound shows hepatomegaly with scattered micronodular liver infiltration.

A first abdominal ultrasound showed hepatomegaly with scattered micronodular liver infiltration (Figure 1). Bile ducts were not dilated. Gallbladder was normal without stones. Doppler exam showed signs of moderate portal hypertension with recanalization of the umbilical vein. Liver elastography was normal. The bladder was distended, but the ureter was thin.

Abdominal scan was performed 2 days later after IV rehydration and revealed a stercoral colitis with a mild peritoneal effusion, with heterogeneous hepatomegaly.

Given these elements, the main etiological hypothesis was a sepsis of gastrointestinal origin with hepatic microembolisms and peritoneal effusion, even if no microbiological pathogen was found despite multiple investigations in the urine, blood, or feces. This caused paralytic ileus and functional urine retention.

Our patient received IV fluids for rehydration and antibiotic treatment (cefepime + metronidazole). A bladder catheter was inserted to manage the distended bladder. Tacrolimus dose was reduced to 0.3 mg/12 h with a target trough level of 5 ng/mL. The azathioprine dose was also reduced to 20 mg/d regarding the bicytopenia and the 6-TGN and 6-MMP analysie. Antibiotics were switched to oral ciprofloxacin and metronidazole after 10 days of IV treatments, with a total duration of 21 days.

Clinical and biological evolution was satisfactory within a week. Apyrexia was obtained after 3 days in parallel with inflammatory syndrome decline. Urinary retention, induced by the initial abdominal pain, quickly disappeared. Serum creatinine was back to normal value (i.e., 57 μmol/L) 7 days later.

However, liver lesions (hepatomegaly with scattered micronodular liver infiltration and alterations of liver function tests) persisted 2 months later.

**Figure 2:**
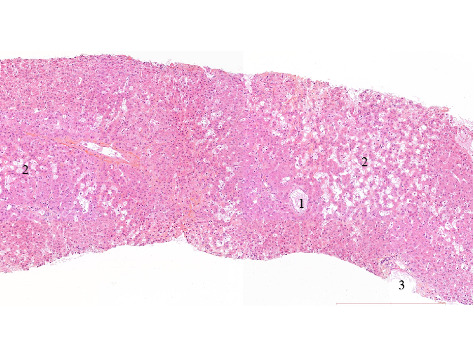
Histological pattern of hepatic peliosis. 1. Normal centrilobular vein. 2. Hepatic peliosis (i.e., sinusoidal dilatation randomly distributed). 3. Portal venous system.

**Figure 3:**
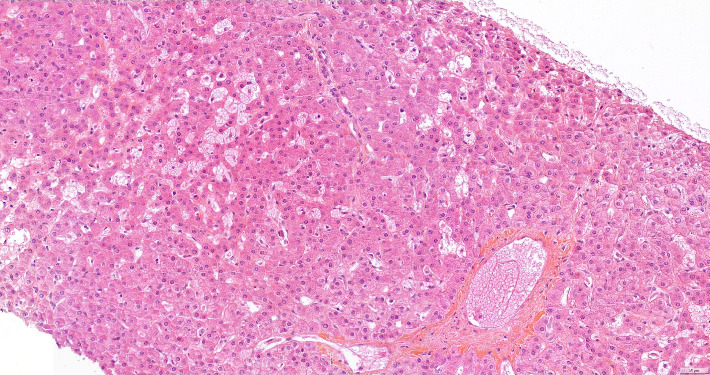
Histological pattern at higher magnification. Parenchymal peliosis is visible, with hepatocytes as lined cells of hepatic peliosis cavities.

A hepatic biopsy was therefore performed. Histopathology study found nodular regenerative hyperplasia with severe parenchymal peliosis (Figures 2 and 3). This hepatic peliosis was attributed to azathioprine, which was interrupted and switched to mycophenolic acid.

The patient did not have a control hepatic biopsy, given therefore no information about the persistence or not of the histological liver lesions. One year after, control sonography found a persistent nondysmorphic micronodular hepatomegaly, and biological liver tests were unchanged. But splenomegaly and portal hypertension had resolved. At last follow-up 4 years later, liver tests were normalized.

## 2. Discussion

Hepatic peliosis is a rare condition consisting of multiple, randomly distributed, blood-filled cavities, without central venous occlusion. First described in the 19th century by Wagner, it was then named “peliosis” by W. Schoenlack in the early 20th, in reference to the Greek word “pelios” meaning livid or bluish [1].

Various causes have been described, such as infectious origin (more specifically with Bartonella infections in AIDS patients and tuberculosis), malignancy, psoriatic arthritis, antiphospholipid syndrome, and drug-induced forms [2–8].

Physiopathology is still partially unknown, and authors usually describe two types of peliosis, depending on the lined cell of hepatic peliosis cavities: parenchymal type and phlebectatic type.

In the parenchymal type, hepatocellular necrosis leads to the subsequent formation of blood-filled cavities. In the phlebectatic type, peliosis could be the consequence of an alteration in the liver blood outflow, causing damages to the sinusoids walls, and then an obstruction in the junction zone between sinusoids and centrilobular venous and therefore sinusoidal dilatation. For some authors, peliosis could be a variant of veno-occlusive disease of the liver and Budd–Chiari syndromes, in which the blockade is located respectively on the centrilobular veins and on the main hepatic veins [9, 10].

Clinical presentation is wide, from asymptomatic cases with only alterations of the liver enzymes and/or hepatomegaly, to severe ones with complications of portal hypertension or hemoperitoneum.

Its long-term outcome and the reversibility remain uncertain, even after interruption of the causal treatment. Lesions can completely decline, stay stable, or lead to perisinusoidal fibrosis. Some authors have also described severe long-term complications such as hepatopulmonary syndrome [11].

Several drugs have been reported as causes of hepatic peliosis: estrogens and oral contraceptives, anabolic androgenic steroids, and azathioprine [3, 7, 8, 10, 12–14].

In their series of 500 patients (aged 16 to 56 years) who received kidney transplantation between 1965 and 1975, C. Degott et al. had identified 55 patients with liver alterations (i.e. hepatomegaly, ascites, and increased level of transaminases), of whom 12 patients had hepatic peliosis (2.4% of all patients). After ruling out the classic known causes of hepatic peliosis, the authors identified azathioprine as the most probable causative agent because all patients received this treatment. This hypothesis was strengthened by the fact that azathioprine had also been incriminated in veno-occlusive disease of the liver or sinusoidal dilatation, both liver diseases with histological patterns closed to hepatic peliosis. To our knowledge, this is still the biggest cohort of kidney transplant recipients with this disease reported so far.

Nowadays, azathioprine is not used anymore as the first-step treatment in kidney transplant by most teams (probably explaining the very small amount of reports of hepatic peliosis in kidney transplant recipients in PubMed since the 90s), but it is still a good alternative drug in case of gastrointestinal side effects linked to MMF.

In conclusion, hepatic peliosis is a rare but known complication of azathioprine treatment, which can be suspected in case of alterations of the liver enzymes, hepatomegaly, clinical, or sonographic signs of portal hypertension, after ruling out infectious causes. The diagnosis is confirmed by histological examination with multiple blood-filled cavities with random distribution. Its long-term outcome and the reversibility remain uncertain, even after interruption of the causal agent.
